# Control and Optimisation of Power Grids Using Smart Meter Data: A Review

**DOI:** 10.3390/s23042118

**Published:** 2023-02-13

**Authors:** Zhiyi Chen, Ali Moradi Amani, Xinghuo Yu, Mahdi Jalili

**Affiliations:** School of Engineering, RMIT University, Melbourne, VIC 3001, Australia

**Keywords:** smart meters, smart grids, control, optimisation

## Abstract

This paper provides a comprehensive review of the applications of smart meters in the control and optimisation of power grids to support a smooth energy transition towards the renewable energy future. The smart grids become more complicated due to the presence of small-scale low inertia generators and the implementation of electric vehicles (EVs), which are mainly based on intermittent and variable renewable energy resources. Optimal and reliable operation of this environment using conventional model-based approaches is very difficult. Advancements in measurement and communication technologies have brought the opportunity of collecting temporal or real-time data from prosumers through Advanced Metering Infrastructure (AMI). Smart metering brings the potential of applying data-driven algorithms for different power system operations and planning services, such as infrastructure sizing and upgrade and generation forecasting. It can also be used for demand-side management, especially in the presence of new technologies such as EVs, 5G/6G networks and cloud computing. These algorithms face privacy-preserving and cybersecurity challenges that need to be well addressed. This article surveys the state-of-the-art of each of these topics, reviewing applications, challenges and opportunities of using smart meters to address them. It also stipulates the challenges that smart grids present to smart meters and the benefits that smart meters can bring to smart grids. Furthermore, the paper is concluded with some expected future directions and potential research questions for smart meters, smart grids and their interplay.

## 1. Introduction

Power systems are among the most critical infrastruture over decades, where the supplied power from large central power plants are delivered to consumers through transmission and distribution networks. To provide high power quality, network operators have been using services from generation units such as frequency and voltage control and black start [1]. However, increase in the electricity demand as well as the social and political movements towards using renewable energy resources to reduce greenhouse gas emissions have made the optimization of power grid a hot topic again in both academia and industry [2]. To address the demand rise using the traditional paradigm, significant capital investments and operational costs are required to upgrade the infrastructure, which will be reflected in higher electricity bills of consumers [3]. On the other hand, renewable resources are uncertain and intermittent in nature and are not as reliable as traditional generation units to support the grid with services such as frequency and voltage control. The challenges have become more serious in the recent decade due to significant penetration of renewable based generation units into power distribution grids. Solar rooftop panels have been cheap enough to be installed in each household, converting traditional consumers to prosumers, i.e., entities which can both generate and consume electricity. With a high penetration level, these distributed generators (DGs) may deliver the whole demand locally at specific times of a day, causing negative demand in the area. This phenomenon has never been experienced in traditional power grids. These challenges, combined with the lack of visibility and situational awareness associated with the obsolete infrastructures, push the power grid to be more vulnerable to frequent disturbances, which often lead to cascade failures or even blackouts [4].

Advancement in sensing and communication technologies have come to help network operators to tackle these issues [5]. Conventionally, grid operators have had limited real-time information on the distribution grid. Fossil-fuel based generation units have been also more controllable and predictable than renewable-based peers [6]. In such a complicated environment, the concept of a ‘smart grid’ has been established to improve resilience and reliability of the modern renewable-based power grid by augmenting communication and control facilities to the system [7]. It enables the intimate cooperation between power systems and information and communication technology (ICT), which guarantees an intelligent and efficient balance between production and consumption [8]. Generation, transmission and distribution networks have been integrated in this concept, and the supply and demand at all levels can be monitored, forecasted and controlled in real-time.

Optimal design and operation of smart grids require real-time information from smart meters. These new entities in the power system have both measurement and communication facilities to measure important parameters, such as power import and export from/to the grid, and communicate it with appropriate data centers [9]. Using these data and advanced data-driven algorithms, the data center can perform decision making algorithms and control the grid. For instance, the demand response (DR) is achieved by merging prosumers and intelligent devices using smart meter functionality. Smart meters may contain extra capabilities such as protection, phase measurements and automation to provide higher flexibility [10].

The installation of smart grids has been undertaken in more than 50 countries. In Australia, the government is promoting more incentives to improve smart grids investments. It also establishes demand-side regulation and time-of-use tariffs and claims that the top priorities are the demand side management, energy security and energy efficiency [11]. In the US, the department of energy has produced “GRID 2030”, which foresees a national vision for the roadmap development of smart grids [12]. The European commission has developed an electricity grid initiative which establishes a nine-year program for researching and developing smart grid technology and market innovations [13]. At the same time, the installation of smart meters is also rapidly growing worldwide. For instance, the European Union countries are required to adopt 71% smart meters by 2023 [14]. The smart meters in the United Kingdom are designed to measure the system parameters every 30 min [15], whereas the rate is doubled in Texas at every 15 min [16]. The measuring rate of smart meters is expected to rise dramatically in the near future to prepare for higher requirements of operating efficiency under the growth of renewable energy integration and a more complex energy market [17].

The research on smart grids and smart meters have attracted a lot of attention in recent years [18]. In particular, the communication architecture and standards for smart grids and the analysis of smart meter data have been highlighted as the key elements to build up future energy systems [19,20,21,22]. Applications based on smart meter data have been developed to satisfy different stakeholder interests [23]. The cybersecurity of smart grids has also been surveyed in [24], which points out that the weakness of communication may cause system data to be exposed to security threats. Although there are some review papers related to control and optimization of smart grids using smart meter data [22,23,24,25], the existing reviews are not as comprehensive as this paper. In addition, the existing papers do not specifically reveal the relationship between smart grids, smart meters and their responsibility of supporting high penetration of renewable energies. In addition, the implementation of smart grids and installation of smart meters are often restricted due to lack of motivation considering limited revenue and privacy concerns. The developments are facing challenges of finding appropriate applications to overcome such drawbacks. Therefore, it is important to find potential applications that promote the power grid to adopt the changes in the penetration of renewable energy and encourage the utility to increase the operation efficiency.

**Figure 1:**
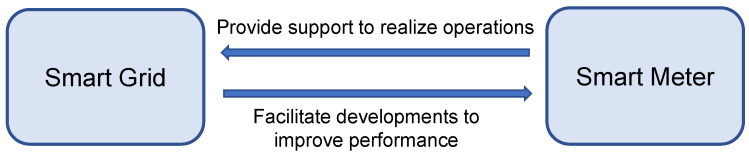
The relationship between smart grid and smart meter.

This paper attempts to provide a comprehensive review of the control and optimization of smart grids using smart meter data, highlighting the research within the last five years. Some earlier classical works are also included. The references are examined through several criteria, including papers that are dedicated to existing and emerging smart grid and smart meter applications, the latest communication and computation technologies, current smart grid developments and investigations of customer awareness of smart meter implementations. The future developments and research direction of smart grids and smart meters can be provided. In this paper, we review the development of applications in the point of views of both smart grids and smart meters, shown in Figure 1, and explain how they support the smooth energy transition towards the renewable energy future. The main contributions are listed below.

1. First, it identifies the challenges that smart grid development faces at each domain level. Then, a review is conducted to introduce applications that aim to solve the challenges. Challenges and applications from smart grid perspective are mainly established from the grid operator side, which aims to increase the profit of the smart grid operations.
2. As the smart meter is essential to support smart grid operations, the challenges of smart grid development are reflected in the smart meter perspective. The review identifies the challenges and provides the applications for solutions. The challenges and applications from smart meter perspective are mainly developed from the customer side, which focuses on improving user satisfaction and smart grid performance.
3. The potential future applications and research directions for smart grids are outlined, which bring about the corresponding future developments of smart meters. The development of smart grids facilitates the evolution of smart metering technologies, while the improvements of smart meters enhance the operation performance of smart grids. In addition, the acceptance of the smart meter implementation is discussed from the customer perspective, which is fundamental for the smooth development of smart grids.

Section 2 demonstrates the concepts of smart grids and smart meters, where the features and capabilities are also described. Section 3 illustrates the detailed control and optimization applications in smart grids using smart meter data, which are demonstrated for both smart grid and smart meter perspectives, respectively. It further explains the interplay between smart grids and smart meters. Section 4 describes the future developments of smart grids and the key challenges to smart meters. It also suggests potential solutions. Section 5 provides the conclusion of the paper.

## 2. Smart Grids and Smart Meters

This section introduces the concepts of smart grids and smart meters. It first demonstrates the definition of the smart grid, including its functionalities and domains, in Section 2.1. The functionalities broadly cover the abilities of the smart grid, where the applications introduced in Section 3.1 are based on them. The domains indicate the fundamental components of the smart grid, and their interactions formulate different smart grid operations. In addition, the impact of the energy transition towards renewable energy on smart grid is introduced at the domain level, which also provides the challenges that the applications are developed to solve. Later, the smart meter and the metering functionalities are introduced in Section 2.2. As smart meters are essential for many smart grid applications, the challenges that the smart grid faces are also reflected from the smart meter perspective. Such challenges provide a guideline for the development of smart meter applications, which will be demonstrated in Section 3.3.

### 2.1. Smart Grids

**Figure 2:**
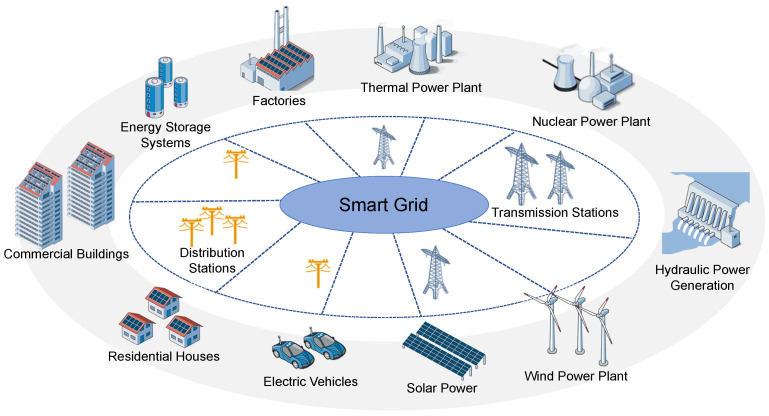
The smart grid framework [26,27].

The commonly accepted definition of a smart grid is an intelligent electrical network that integrates information, two-way cyber-secure communication technologies and computational intelligence throughout the energy system from power generation to consumption endpoints [12]. Several state-of-the-art communication technologies, multi-tariff meters and power distribution devices have been applied to guarantee the efficiency and reliability of the operations through energy generation, transmission, distribution and consumption. A network model of a smart grid is shown in Figure 2.

Generally, the smart grid has multiple sensors to support remote testing and coordinative control, which enable self-monitoring and self-healing capabilities. It also features with auxiliary functions including the integration of renewable and distributed energy sources and the data exchange among renewable energy sources (RES) and electric vehicles (EV) [7]. Each function of smart grid contains a number of specific technologies that cover the whole grid, from generation through transmission and distribution, to a variety of consumers. The key functions are demonstrated in Table 1.

**Figure 3:**
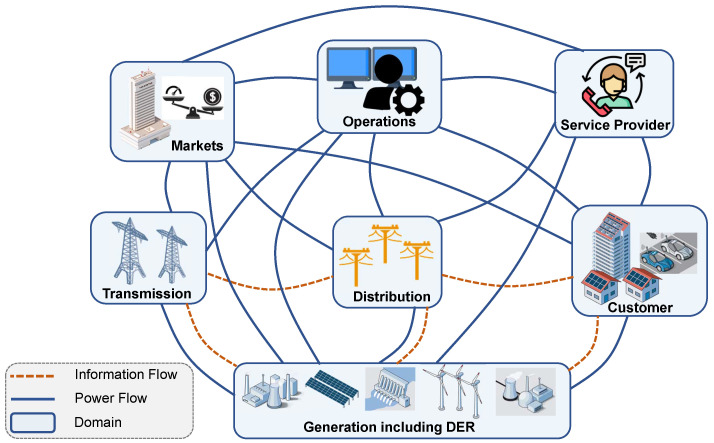
The conceptual model of the smart grid [28].

According to the national institute of standards and technology (NIST), smart grid can be separated into several domains, each of which consists of many components and applications [28]. The domains are shown in Figure 3.

#### 2.1.1. Energy Generation

This domain focuses on power generation to supply the consumers’ demands. It connects to the power transmission and distribution domains through electrical and communication networks. The communication network exchanges information on generator performance, as well as quality-of-service issues such as scarcity and generator failure [29]. It also enables the routing of electricity from multiple power sources due to lack of sufficient supply [30]. With the development of power girds, the power generation domain faces new challenges such as reducing carbon emissions and increasing the use of RES [8]. In addition, the distributed energy resources (DER)s significantly contribute to the power generation in smart grids, improving the capacity and flexibility of smart grids, yet, leading to higher operation complexity.

#### 2.1.2. Power Transmission

The domain focuses on high voltage power delivery over long distances. As power transmission is the boundary of generation and utilization, it usually operates in substations. The substations are responsible for levelling-up or levellinv-down the voltages for connecting the generation and distribution, which contain control and protection devices. A supervisory control and data acquisition (SCADA) system is used for monitoring and controlling the transmission network via communication networks and regulation devices [31].

#### 2.1.3. Power Distribution

The domain is responsible for providing electrical interactions among distributed generation (DG), distributed energy storage system and consumer consumption. The reliability of the distribution system depends on the types of devices and their communication structures. The operators need to balance the initial investment and reliability when choosing the network structure among various categories, such as looped, radial or meshed [32]. In addition, the devices closely communicate with the operating system in real-time to regulate power flows. The regulation regards the dynamics of power demand, considering factors like market value, environmental impacts and security [33,34]. The interaction with the market will affect localized consumption and generation, which would further influence the development of distribution system, or the even larger utility grid, from both the structural and electrical perspectives.

#### 2.1.4. Operation

The operation domain is responsible for managing the electricity delivery with reliability and efficiency under different situations. The operating systems are essential through power generation, transmission and distribution to the consumers. Many applications are developed in this domain in terms of monitoring, control, demand side management, fault management, operation planning, maintenance and consumer support [35]. The main challenge for the operation domain is to improve the overall performance of such applications and provide innovative ones for adopting the future renewable energy transition.

#### 2.1.5. Utility

The utility domain is responsible for supporting the business process among power generators, distributors and consumers. The utility ranges from conventional applications, like billing and consumer account administration, to advanced applications, like energy management and home power generation [36]. It is connected with the operation, consumer and market domains. The communication with the operation domain is essential for situational awareness and system regulation. The connections with consumer and market domains are necessary to encourage economic growth with a variety of applications in smart grids [37,38,39]. The utilities produce novel applications to match different requirements and opportunities introduced by the development of smart grid. The main challenge for the utility domain is to provide a core interface and standards to support the active power trading market, while maintaining operation stability of crucial power infrastructures. The interfaces are required to support different types of communication technologies, while maintaining high quality connections.

#### 2.1.6. Electricity Market

The market domain balances the electricity supply and demand with respect to the market price. The participation of DERs in the power market makes the smart grid more interactive, which in turn, brings about more data exchange among different domains [40]. Therefore, their communication is required to be reliable, efficient and traceable. The communication latency should be reduced as the operation rate rises [9]. The challenges in the market domain are the expansion of DER information to each community subdomain, expanding the interactions of utilities and consumers, simplifying the market rules and developing interaction frameworks for regulating the rising retail and wholesale energy trading markets.

#### 2.1.7. Consumer

The consumers are the end-users of the electricity, who are the stakeholders that the utility domain is developed to support. The consumers manage their energy consumption and generation, which electrically connect to the distribution and generation domains through smart meters. The consumer domain communicates with market, operations, distribution and utility domains, where the smart meters provide a bridge to operating systems, like building automation systems or energy management systems [41]. Generally, the consumer domain is separated into subsystems with respective to home, building, commercial and industrial. Each subsystem has several applications, including the display of customer power usage, remote monitoring and regulation of DGs and loads [42,43,44,45]. The main challenge in the consumer domain is to improve customer awareness and acceptance of smart meter implementation.

The smart grid promotes higher automation and control capabilities to the transmission and distribution grids; it also adds more complexity to the power system, which challenges the reliability and safety of operations [17]. More requirements of energy measuring units are proposed to realize the smart grid operations, which shape the development of smart meters.

### 2.2. Smart Meters

**Figure 4:**
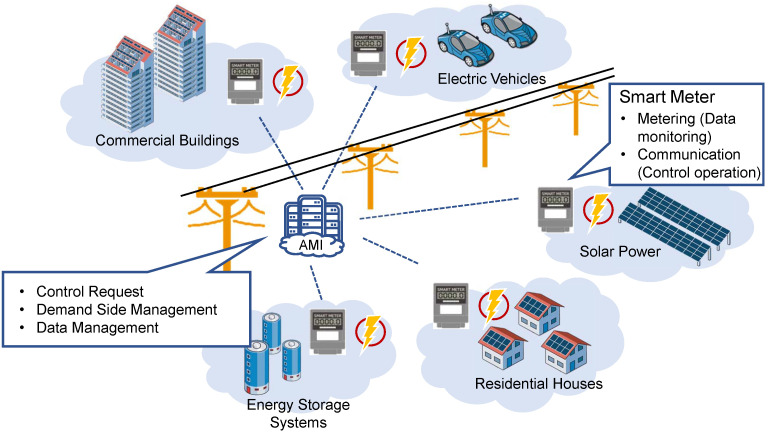
Smart metering configuration and functionalities.

Smart meters are energy measuring units that locally record a number of electricity parameters of each prosumer, such as power consumption and power export, and sends the data to a central server through communication networks for operation purposes. A common smart metering system employs metering and communication infrastructures, which is shown in Figure 4. The metering infrastructure allows the smart meter to have functions such as voltage and current waveforms recording and data storage. The communication infrastructure enables the bidirectional communication between consumer and utility through the power line connection or wireless connection. It allows the smart meters connect to the remote centers for control and management purposes, which forms an advanced metering infrastructure (AMI) [41]. The evolvement of smart metering systems is correlated to the development of smart grids, which is reflected in several features.

#### 2.2.1. Periodic and Precise Metering

The basic function of a smart meter is to perform regular and accurate measurements of electrical parameters such as voltage, current, power, frequency and power factor [46]. These parameters are essential factors for load management, load profiling, load monitoring and fault analysis. The monitoring and recording activities are activated at particular time intervals, where the frequency varies from a few minutes to a few hours [9]. Due to the high level of intermittence and uncertainty in both generation and demand sections, especially in the presence of RES-based generation units, reliable operation of smart grids requires more real-time actions compared to conventional power systems; thus, the frequency of data collection in a smart grid is high. This high periodic measurement provides a more detailed understanding of system operating patterns, which benefits applications like load forecasting and demand response. In contrast, it also adds challenges to smart meters, such as the deployment of the smart meter in a way that can achieve the most efficient measurement, and how to collect data in an efficient way.

#### 2.2.2. Data Collection, Storage and Alarming

In smart grids, the increased data measuring frequency greatly increases the amount of measured data. Since the current computational capacities are often not enough to process the whole dataset, efficient data extraction is required to support effective decision-making processes [47]. In addition, smart meters can store the measured data to prevent data loss when the communication to the central server fails. The data can be locally collected from meters, which adds to the reliability of the system. The stored data can benefit the energy suppliers and consumers to track the history of energy consumption. Billing and cost data can also be stored for further reference and support decision making. Furthermore, the smart meter can make alerts and notifications based on a variety of alarm conditions on different monitored parameters. These alarms are beneficial for fault-preventing applications such as fault detection, energy theft prevention and system security [48,49]. The challenges for this feature are effective data extraction and accurate fault detection using measured data.

#### 2.2.3. Communication Interfaces

There exists a bi-directional communication between smart meters and the grid operator, that allows operators to provide reliable operation, better maintenance, optimal demand management and to efficiently plan for the infrastructure upgrade and expansion [50]. It also allows consumers to track their energy usage, take part in the demand management programs that the operators run, and have revenue from selling electricity to the grid [20]. The communication is achieved by using either wired or wireless technologies [19]. The practical connection often adopts hybrid communication, where the advantages of different technologies are exerted under various situations to meet the communication requirements. The challenges for this feature are avoiding communication traffic and keeping efficient communication, while achieving optimal levels of reliability and security.

#### 2.2.4. Demand Side Management

As the two-way directional communication allows the smart meter to receive remote control instructions, it has the ability for consumers and energy suppliers to regulate the power consumption, yielding more efficient use of resources in power systems. For instance, the consumer could turn off the thermostatic home appliances such as air conditioners at peak demand intervals, when the bill is the highest, and turn them on for the rest of the intervals. The energy suppliers could have more opportunities to reduce the total power usage in peak periods by sending instructions to smart meters. These concepts are regarded as demand side management [51], which has the fundamental rule to redistribute the power consumption. Consumers are motivated to switch their power usage from peak periods to valley periods, according to dynamic pricing [52]. The challenge for this feature is increasing the reliability and accuracy of demand side control, while keeping the consumers’ satisfaction within a tolerable level.

#### 2.2.5. Data Management Systems

Smart meter data management allows the smart grid to apply analytical tools for processing data to achieve objectives such as improving and optimizing utility grid operation and management [22]. It also provides references to decision making for the development of smart grids [53]. The challenges for this feature are improving more accurate data analysis results in shorter time periods and developing proper algorithms to support the implementation of novel smart grid applications.

## 3. How Smart Meters Support Smart Grids

**Figure 5:**
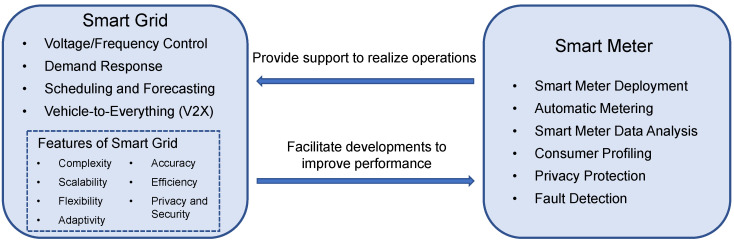
Applications from smart grid and smart meter perspectives.

The interrelated roles of smart meters and smart grids can be shown in Figure 5. From the smart grid perspective, the applications are mainly focused on how smart meters can support the coordination of different electric devices to realize a reliable power system. On the other hand, these applications aim to improve the performance and efficiency of smart metering. Such aims reflect the features of smart grids, which promote the development direction of smart meters.

### 3.1. Applications from Smart Grid Perspective

The development of smart grids had let to a large number of coordinated control and optimization algorithms covering a variety of domains with multiple aims. The traditional approach of power systems has to be improved, while taking into account challenges such as system complexity and flexibility. This subsection introduces how the smart meter can help to realize devices coordination in smart grids aiming for different objectives [54].

#### 3.1.1. Frequency and Voltage Control

Frequency and voltage regulation are the two most common operations in power system stability and control [55]. The frequency must be maintained to close to the nominal value for the integrity of infrastructure and system protection [56]. The voltage magnitude must remain close to its rated value to guarantee the effective behavior of end-users’ equipments and to prevent system collapse that may lead to blackouts [57]. Both control objectives are required to be achieved efficiently while considering their specific features. In particular, the voltage and frequency control are achieved by considering the power management that can enhance the system stability, while curtailing power losses. The voltage and frequency drop can be mitigated by minimizing the active and reactive power flows, respectively.

In smart grids, voltage and frequency control are performed much more flexibly, due to the deployment of DG units. With the implementation of smart meters, the smart grid has the capability to realize real-time voltage and frequency measurement, and bidirectional communication between consumers and network controllers. It further integrates the conventional large-scale power plants into grid systems, which contain thousands of distributed generators such as PV systems and wind turbines [13]. The grid systems can either operate in grid-connected mode or islanded mode, where bidirectional power flows are enabled among prosumers under various power conversion technologies [58]. The installation of smart meters allows power conversion operating in distributed control schemes, where centralized control is avoided to prevent problems like single point of failure or high control cost under a large number of DGs [59]. Moreover, the power network data collected from smart meters improve the operating performance of smart grids. As the distribution system of smart grids is becoming more complex, the system operators often lack exact knowledge of network parameters. To maintain performance, the operators apply data-driven approaches to accurately find system behaviors based on smart meter data [60]. For instance, the network status measured by smart metering technologies is analyzed and used to generate decision making for various control devices like on-load tap-changer (OLTC) to solve problems like overvoltage and undervoltage across the whole network [61]. In addition, the installation of smart meters provides consumers with more suitable individual energy services with the aid of model predictive control (MPC). It uses measured information such as individual energy consumption behaviors to predict future control decisions [62].

#### 3.1.2. Demand Response

**Figure 6:**
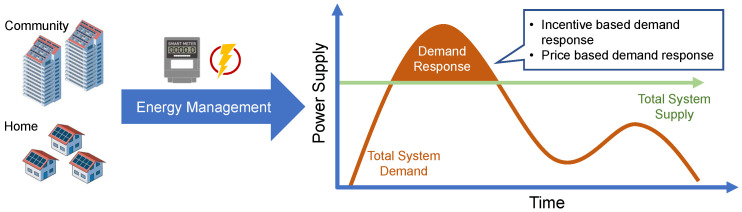
Demand response configuration.

The evolution of smart grids inevitably brings about the increasing penetration of renewable energies and installation of energy storage systems. The demand side management strategies are hence introduced to solve problems such as high generation cost, high demand peak-to-average ratio and transmission congestion, while avoiding reduced service experiences of consumers [33]. A basic configuration of demand response is demonstrated in Figure 6. The peak demand impacts the grid flexibility and may break the balance between energy supply and demand [63]. Therefore, the basic idea to achieve demand side management is to change the energy consumption behaviors of users from their normal consumption patterns, by using either price based or incentive based stimulations to maintain the system reliability [64]. The incentive based demand response aims to reduce the consumers’ energy usage at peak hours and provides considerable incentives in return [65]. The price based demand response offers different electricity prices at different times, where the energy consumption behaviors of users are changed accordingly [66]. Applications of demand side management are mainly developed on home energy management and community energy management, where the smart meters are the core components to integrate a large variety of electrical devices for coordination through online functionalities such as energy usage data measuring, analyzing and reporting. The integration increases the capacity of demand response under large power fluctuations. With the installation of smart meters, consumers are more aware of their energy usage from time to time, which further promote the realization of demand side management. For instance, the increasing number of electrical vehicles adds more pressure to peak demands, particularly in residential areas, where electrical vehicles cause high demand loads. However, consumers are likely to shift their charging and discharging behaviors for lower prices, and smart meters are the key component to facilitate such load shifting [67].

Energy storage systems are often applied to accommodate fluctuations caused by renewable energies and control the power peaks in residential areas. Sufficient energy storage capacity is the prerequisite that allows smart grid operators to provide demand response, while the capacity is also positively correlated to its cost. In addition, the placement of energy storage systems is essential for optimizing the energy efficiency, as well as minimizing the fundamental investment. The properly placed and sized energy storage systems benefit many grid operations such as peak demand shifting, integration of renewable energy sources, power quality enhancement and transmission cost reduction [68]. To facilitate the optimal placement and sizing of energy storage systems, various types of power network data are essential to be collected prior to analysis and modeling, through a variety of optimization algorithms [69].

#### 3.1.3. Scheduling and Forecasting

**Figure 7:**
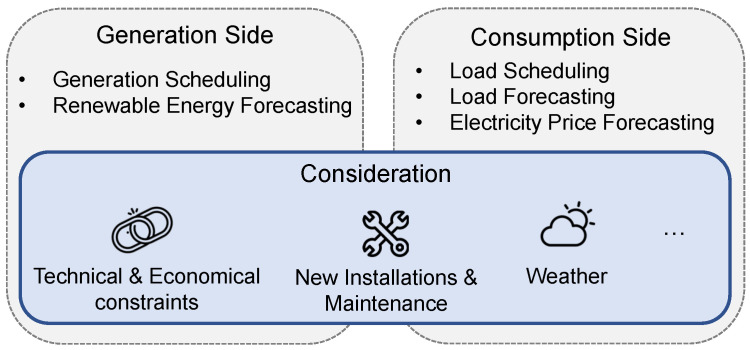
Scheduling and forecasting.

During the operation of smart grids, various flexible power generations, energy storage systems and load demands are involved. The utility operators require scheduling and forecasting to provide constant, sustainable and rapid reserve supply in the power grid. In addition, the increasing penetration of renewable generations brings about additional uncertainties. Operators require more information for decision making for more frequent changes between supply and demand. Generally, the scheduling and forecasting techniques are applied on both the generation and consumption sides to guarantee the reliability of smart grid while maintaining its flexibility. An infographic is shown in Figure 7. From the generation side, the generation scheduling applies fast reserve supply, which requires the resources generated by the reserve with higher resolution to provide reservation and balance uncertainties of wind and solar power plants through demand response [34]. In addition, renewable energy forecasting drew much attention as it improves the certainty of the power generations [70]. However, the forecasting is based on data driven techniques, which require accurate and sufficient system information and efficient algorithms to ensure the performance [71]. From the consumption side, load scheduling and forecasting are often applied, where the smart meter is used to identify shiftable and non-shiftable loads to facilitate the demand response. It also records relevant information in detail and helps to generate precise scheduling model of the smart grid. With the aid of novel learning techniques, accurate load forecasting, considering technical and economical constraints, is achieved [72]. In addition, the uncertainty of weather also increases the complexity of electrical load forecasting. For instance, the value of thermal loads is highly related to the local temperature. Smart metering technologies can collect and process the nonlinear data and obtain accurate load forecasting [73]. As the smart grid is becoming more complex and more prosumers are involved in the distribution side, power trading markets are becoming more active. The electricity price forecasting has drawn more attention due to its fundamental influence on the decision making process of utility operators [74]. The price forecasting also faces challenges, as the integration of renewable generations and energy storage systems often increases its uncertainties. Such uncertainty could be solved with the support of high resolution smart meter data, where precise price forecasting is made from the short-term to the long-term [75].

#### 3.1.4. Vehicle-to-Everything (V2X)

The term “V2X” indicates the applications that focus on vehicle-to-everything, which allows electric vehicles to connect with other objects such as other vehicles, infrastructures and customers. Nowadays, electric vehicles are becoming more popular due to the increasing gas price and more consideration of carbon emissions. Reports indicate that the number of electric vehicles has reached 13 million in 2021 and is expected to exceed 73 million by 2025 [76]. With the increasing use of electric vehicles, their energy capacity has grown to a considerable number that is required to be integrated in the demand response. The installation of smart meters realizes such integration, where the electric vehicle users in their smart homes become prosumers. They can make choices to reduce their electricity cost according to the optimal scheduling algorithms. Smart meters provide incentives, like current electricity prices, to help prosumers decide the change or discharge of their electric vehicles from time to time [77]. Electric vehicles are hence switching between home-to-vehicle mode (charging) and vehicle-to-home mode (discharging), to interact with the smart grid. The integration of electric vehicles is operating along with stationary energy storage systems to compensate for the fluctuations caused by renewable energy generations and loads in the grid [78]. On one hand, the smart meter data reflect the charging and discharging behaviors of electric vehicles, which support the scheduling and decision making for utility operators through data driven approaches [79]. On the other hand, the smart meter data also provide detailed information of the vehicle’s battery, which is closely related to the battery’s state of health. In particular, effects such as ageing and degradation impact the battery capacity and performance, which further influence the service experience of prosumers [80]. To effectively maintain the battery lifetime, prosumers make predictive maintenance services through prognostic methods [81].

### 3.2. Features of Smart Grid

**Figure 8:**
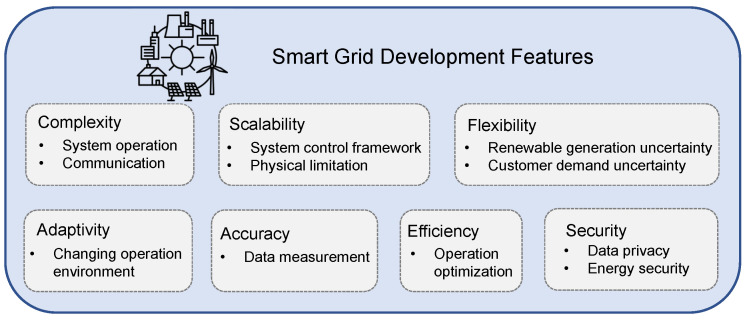
Features of the smart grid.

The smart grid is developed toward a more complex power grid to meet an increasing number of stakeholders. Its evolution reveals several features, shown in Figure 8. These features become the design requirements to facilitate the evolvement of smart meter applications.

#### 3.2.1. Complexity

Complexity is one of the most essential features of smart grid, as it involves numerous electric devices and systems mutually coordinated at multiple voltage levels under various time scales. The growing penetration of renewable energy and increasing load demand lead to more complexity in dealing with the increasing uncertainties [50]. The complexity of smart grid reflects the complexity of smart meter communication, which in turn, also challenges the configuration of communication networks among smart meters.

#### 3.2.2. Scalability

The development of the smart grid brings about high penetration of DG units, which are distributed throughout the power systems, particularly at the distribution domain. The control of such units requires a large number of controllers, which challenges the current scheme of power system regulation. Recently, distributed and hierarchical control approaches have been implemented to solve such issues [82]. The units achieve the control and optimization objectives by communicating with their neighbors or higher level units, where the connections are usually realized by their locally equipped smart meters. The improper communication among smart meters may deteriorate the operating performance of smart grids. The coordinating strategy is required to be optimized, considering the physical limitation of communication and system constraints [83].

#### 3.2.3. Flexibility

Flexibility of the smart grid indicates that it is able to adapt variability and uncertainty using available resources during a certain time period in an economic approach [84]. It allows the power system to efficiently accommodate the uncertainties from wind/solar generation and end-users’ demands with various distributed energy resources and flexible loads. The coordination among various devices are often flexible changes in different time and regions, where the smart meters are required to provide sufficient and timely network data for support [85].

#### 3.2.4. Adaptivity

The adaptivity indicates that the controller could update its parameters according to the changes in the controller units [86]. In smart grids, the dynamics of the electricity market are becoming more active due to the significant increment of various market players, different types of DG units and their interactions. The changeable behavior requires controllers to adaptively adjust the control inputs, so that the system can remain stable [87]. In addition, the topologies of power networks also require such features considering cyberattacks. The connection may not be available facing an attack or a failure, while the system stability should be maintained. The uncertainties are often dealt with through proactive and online strategies [88].

#### 3.2.5. Accuracy

Smart grids require high accuracy to reach complex coordination among multiple energy resources and loads. The accuracy stands for both the time instant perspective and the system parameter perspective. In particular, smart meters are required to provide real time system measurement and more frequent communications to allow complex system coordination in smart grids [89]. However, such requirements challenge the data storage capacity and communication constraints of smart meters [9]. The optimal strategies for smart meter deployment and measurement, as well as data analysis, are necessary to solve such issues.

#### 3.2.6. Efficiency

Considering the limitation of the resources and physical constraints, the smart grid is required to operate with high efficiency. This indicates that optimization techniques are involved to minimize the operating cost [71]. As the complexity of the smart grid is growing, the system is required to transmit an enormous amount of data to achieve accurate and timely operation, increasing the pressure on the communication network. Therefore, approaches are required to extract fundamental information from the measured data before transmitting it through communication networks. Such approaches often taken place at the smart meter, by using certain algorithms such as a singular value decomposition based method [90].

#### 3.2.7. Security

The measured power system data are closely related to the energy consumption behaviors of users. Their privacy concern has draw more attention and has become a substantial obstacle in the development of the smart grid. Therefore, privacy preservation has become one of the crucial features of smart grids to prevent information leakage such as address and identification [91]. Energy safety is another perspective of security, where energy theft and operation fault are crucial factors that influence smart grid performance [92].

### 3.3. Applications from Smart Meter Perspective

#### 3.3.1. Smart Meter Deployment

As the smart grid becomes more complex, it requires massive smart meters for data collection monitoring and regulating utility energy consumption. Conventional approaches require a large scale of energy meter networks, which often suffer from high deployment, maintenance and data collection costs [32]. Thus, the deployment optimization of smart meters has drawn great attention. In particular, the deployment is required to use minimal number of smart meters to track massive utility states on time [93,94,95]. Research works often apply optimization techniques to the communication networks of smart meters, while considering practical constraints. For instance, one approach is to decompose the power distribution network into a forest of several trees, where the deployment locations, as well as the required number of smart meters, are optimized accordingly [32]. In addition, as the operation of smart meters requires energy, the energy consumption of large-scale smart meters is significant. It brings about problems to find optimal deployment and data transmission rate for smart meters to minimize the overall energy consumption [93]. Furthermore, the physical limitations of the communication networks could impact the overall performance of smart metering [94]. Limitations such as communication bandwidth and data transmission rate often result in communication delay and further jeopardize power system stablity [95]. Such constraints should be considered during the optimization of smart meters deployment to obtain feasible results.

#### 3.3.2. Automatic Metering

**Figure 9:**
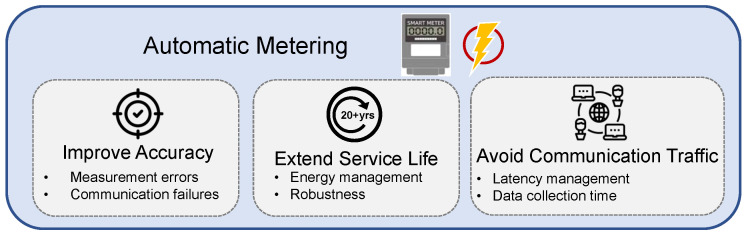
Essential factors for automatic metering.

Since the smart meter is the key element in the control and optimization of smart grids, the approach for smart meters to collect and transmit data with accuracy and efficiency is vital to achieve stable and efficient operations in smart grids. The essential factors for automatic metering are illustrated in Figure 9. The smart meters can be deployed in various situations to measure different parameters. For instance, as smart meters are implemented in the customer domain, load monitoring is one of the important applications to track power usage for energy management. Traditionally, load monitoring is achieved through a power meter at a building level, which is classified as non-intrusive load monitoring (NILM) approach [42]. As the approach provides aggregated data, it is hard to differentiate power consumption patterns for different loads. Therefore, the intrusive load monitoring (ILM) approach is introduced, which provides precise power consumption of individual loads by installing smart power plugs at each electrical socket [43]. The ILM approach can further support applications such as activity recognition [44] and user–appliance interaction [45].

As some parameters in the smart grid are not available for direct measurement, state estimation techniques are implemented to solve such issues [96]. In addition, smart meters require accurate consumption metering, even under extreme operating conditions, which is also a crucial factor to influence consumers’ confidence in using smart meters [97]. The customers require precise energy readings to optimize their energy cost. Therefore, many techniques have been proposed to improve the observability and robustness of smart metering by enhancing the performance of state estimation algorithms against various uncertainties such as measurement errors or communication failures [96,97,98,99]. In particular, state estimation algorithms could apply uncertainty propagation theory to optimize the accuracy performance with the integration of smart meter data at multiple voltage levels [96]. The robustness against uncertainties can also be improved by data driven approaches, where the historical measured data are used for supervised learning to provide accurate system operating patterns [98]. As the smart meter can offer more precise energy measurements for individual loads, more detailed parameters in the power system can be obtained, which in turn, improves the state estimation accuracy [99].

For constantly providing quality services in smart grids, the smart meters are expected to last for several decades. Hence, it is crucial for them to manage their available energy resources. In other words, smart meters need to balance their operational performance and system lifetime according to energy management policies [100]. One feasible approach is to harvest energy from environmental resources such as solar and wind when they are available; otherwise, harvest electricity from smart grids [101,102]. The approach maintains the reliability of smart meters, while solving the uncertainty of environmental resources. In addition, energy harvesting management techniques are designed to consider the performance of monitoring and communication with high efficiency [55].

With the development of smart grids, smart meters interact during multiple operations, and the interaction becomes more frequent, especially in peak hours. This may create a lot of traffic and interference. Such issues lead to data transmission delay, where the latency management of the communication networks can be made [40]. The smart meters can also be separated into clusters with respect to their locations, and a data collection time is assigned based on the communication network [55].

#### 3.3.3. Smart Meter Data Analysis

**Figure 10:**
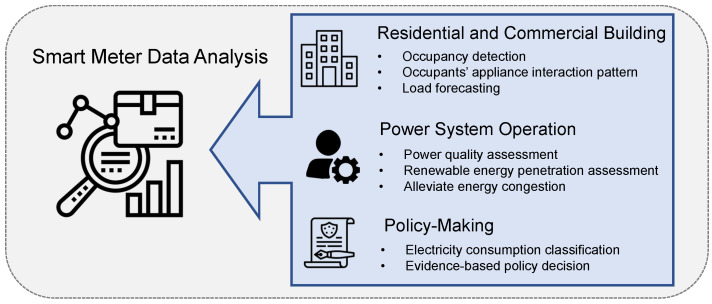
Aspects that smart meter data analysis is dedicated to improve.

One of the advantages of the smart meter implementation is that it could provide more detailed measurement data in power networks with near-real-time resolution. By analyzing the data through cloud computing or machine learning techniques, many applications are proposed to improve the performance of smart grids in various aspects, which is shown in Figure 10.

In the residential and commercial buildings aspect, the smart meter data can be applied to analyze the occupancy status of households. With the aid of machine learning techniques, occupancy detection can find not only the current status, but also predict it in the future [36]. In addition, the occupants’ appliance interaction patterns can be identified by analyzing both the power consumption and occupants’ presence data. The patterns reveal different profiles of interactions between occupants and appliances, which further improve energy performance [45]. For instance, an IoT-based plug load management system is proposed based on occupancy information. The system reduces the power consumption of plug loads (such as laptops and monitors) for individual users through their personalized control preferences, which greatly improves their satisfaction [103]. Furthermore, the smart meter data can feed data-driven models for building-level short-term load forecasting, which benefit demand response applications [104].

For the power system operation aspect, the smart meter data improve power quality assessment and renewable energy penetration assessment [25]. For instance, the growing penetration of renewable generations leads to overvoltage and overcurrent during their operations [105,106]. Analyzing the data measured by the smart meters allow operators to design effective control strategies, as well as protective devices to mitigate voltage and current violations [107]. On the other hand, the data allow operators to alleviate energy congestion during peak hours, considering the physical limitations of power transmission networks [108,109]. Using the data collected among smart meters, the congestion issue can be modelled as a knapsack problem, where the utility control centre can disconnect certain DGs accordingly to maintain the system stability [108]. In addition, as the customers’ behaviors are unknown, the model can be generated based on the Poisson point process to avoid the traffic of power transmission networks and maximize the transmission throughput [109].

On the policy-making aspect, the smart meter data help to facilitate making evidence-based policy decisions. In particular, the smart meter data are closely related to the power consumption patterns of users, which can be used for electricity consumption classification [110]. It reveals demographic and locality information of customers that provide the grid operator with better knowledge of their occupancy behaviors [111]. Such information can benefit demand response operations and potentially support evidence-based policy decisions [112]. A case study to optimize energy management and the required energy capacity for a large group of households in the Netherlands is conducted over one year [113]. The study shows that consumers contribute to the demand response by changing their consumption behaviors according to the dynamic electricity tariff. The data describe the detailed energy consumption behavior with and without energy storage systems, which could further promote the development of novel energy technologies, as well as proper demand response policies.

#### 3.3.4. Consumer Profiling

**Figure 11:**
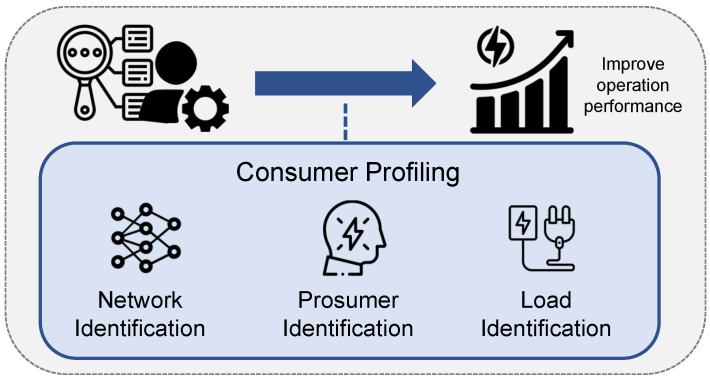
Consumer profiling.

The smart meter data often reflect the energy consumption behaviors of customers, which can be used for analytical studies to identify profiles such as network topologies, loads and prosumers. An infographic is shown in Figure 11. The identification could provide more information to the grid operator, so that the operations could be more precise and, hence, more efficient. In particular, the topology information of a power distribution network is crucial for its efficient operation. However, the network connectivity is often not available in low voltage distribution networks due to its flexibility and complexity. The number of prosumers who consume and produce energy through PV power generation is also increasing. Such prosumers in the networks change their energy production and consumption behaviors from time to time. To identify the distribution network topologies in a cost-effective way, the data driven approach can be used based on smart meter data [37]. The measurement of smart meters provides the network connectivity in time series, where the graph’s theoretic interpretation is applied to infer the steady-state network topology. For prosumer identification, the electricity utilities have lack of knowledge of the location and size of all solar prosumers due to unauthorized or unreported installations. However, the installation of smart meters increases the visibility of energy flow among end-users, which provides granular information that improves the accuracy of prosumer identification and load profiling [38]. Such knowledge can support better circuit protection and voltage regulations and improve the situational awareness of smart grid operators, such that a more efficient demand side management strategy can be applied [39]. The load profiling indicates the categorization of load according to energy utilization behaviors. The uitility operators would improve the scheduling and forecasting processes and arrange proper demand side management if accurate load profiling is provided. For instance, knowledge of flexible loads like air conditioners and washing mechines could support the operator making accurate optimization models for economic dispatch [22]. However, as accuracy of the profiles improves, the data complexity and variability are also increased. It would bring about more pressure on both the communication and computation capacity. To reduce the data size while maintaining their accuracy, approaches such as clustering algorithms are hence studied [114].

#### 3.3.5. Privacy Protection

As the smart meter data reflect detailed energy consumption behaviors of consumers, it leads to serious concerns about privacy protection [24]. There are two main approaches to privacy protection. The first approach modifies the smart meter data before sending it to the utility provider. The fundamental concept of this approach is to distort the data to protect the privacy of individuals, which includes methods such as data obfuscation [115], data aggregation [116] and data anonymization [117]. However, distorting the data also prevents the distribution system operator from precisely monitoring the grid parameters and making rapid reactions. The second approach is to directly modify the actual energy consumption profile by introducing energy storage systems. The actual energy consumption behaviors of users are hidden as the charge and discharge of the energy storage systems are added to the total energy consumption [118]. However, it also impacts the accuracy of data analysis, which further deteriorates the operating performance such as load forecasting and energy storage sizing. The privacy protection is expected to be one of the essential topics of smart grid developing towards cyber-physical systems, where more online interactions are involved among multiple domains in various applications.

#### 3.3.6. Fault Detection

During the operation of smart grids, there are various types of electrical incidences occurs where reactions are required to take place rapidly and effectively. The operators could only achieve this with the support of advanced monitoring and decision assistant tools for collecting and analyzing real-time data from the whole power system [119]. The fault detection is the essential factor to the reliability of the smart grid, which also provides the smart grid with the ability to self-heal and isolate to avoid or limit negative consequences [120]. The faults are often associated with abnormal voltages, currents and phases, which can be detected by analyzing their shapes [121]. Smart meters are key components that measure system parameters such as the temperature of transmission lines, power outages and power usage. They could detect the faults and alert the operators through communication networks. Furthermore, smart meters can be applied to transmission and distribution networks to reduce energy losses, both technically and commercially [122]. The technical energy losses are related to the miss operation of the power system, where the smart meters will alert the operator by applying algorithmic procedures [123]. The commercial loss is often caused by energy theft, where the implementation of smart meters can detect remote exploits and local physical tampering of energy theft. In particular, the smart meters form an intrusion detection system, which combines the energy consumption data with audit logs of physical and cyber events to generate a more accurate model for detecting theft-related behaviors [124].

## 4. Further Developments of Smart Grids, and Challenges of Smart Meters

Future smart grids are expected to operate in more efficient, flexible, reliable, sustainable, decentralized, secure and economic manners [5]. In particular, the smart grid is expected to implement more distributed generators to solve large transmission loss due to long transmission distances. They will be downscaled into microgrids and nanogrids, which have more connections with multiple voltage levels [125]. More electrical devices will participate in energy management through the energy market. More secure data encryption and transmission protocols will be involved in various communications. Such changes will result in more complex and frequent operations in the smart grid. To ensure operational stability, the smart grid essentially requires higher computation and communication capabilities, which in turn, promote the future development of smart meters.

### 4.1. Communication Capacity

A reliable communication network is one of the essential prerequisites to stable operations in the smart grid. In particular, many applications in the smart grids are achieved through distributed approaches, where smart meters are involved to provide a reliable communication network for support. Data collected and transmitted through smart meters realize the coordination of multiple electric devices such as EVs and energy storage systems. An unreliable communication network will impact the coordination and further deteriorate the operation performance of the smart grid. Some studies assume that smart meter data can be transmitted on time [126]. However, in practical smart grid operations, communication delays and transmission errors are often inevitable, which can result in negative impacts on the stability of power systems [127]. To overcome such challenges, the smart grid could apply 5G technologies to speed up the data transfer rate. The 5G technology stands for the fifth-generation cellular network technology, which has been developed and applied in many countries due to its advantages of data transfer speed, reliability and security [128]. In addition, due to the mobility feature of 5G communication, it could further allow consumers to better track their energy usage and participate in the demand response through remote control applications. For instance, the communication between EVs and smart meters allows customers to remotely track and control the charging and discharging of their EVs according to the incentives. The communication also enables the grid operator to obtain the latest information of the overall EV energy consumption and to provide suitable demand response strategies [129]. However, there are also challenges to the implementation of 5G technology due to its low transmission distance. The electromagnetic wave frequency spectrum of 5G is much larger than the frequency of 1G–4G, which brings about a large communication bandwidth, but a lower transmission distance [130]. The ability to transfer data through obstacles is much lower than 1G–4G, which leads to less communication reliability. It brings about challenges to the development of smart grids. To solve such issues, one approach is to increase the density of 5G cellular network. However, this also results in higher deployment cost [131]. The other feasible approach is to apply hybrid communication technologies [132]. The data can transmit among multiple communication protocols in different circumstances. In addition, the energy transfer capacity of 5G is much higher than the 4G, which indicates that more data can be transmitted simultaneously. However, it also has higher energy consumption, which in turn, challenges the energy management strategy on how to optimize data transmission [133]. Furthermore, research on 6G technologies, considering future communication traffic, is proposed, due to the explosive growth of communication demands [134]. Compared to 5G, 6G technologies are superior in multiple perspectives such as data transmission rate, efficiency, reliability and security.

### 4.2. Computation Constraints

With the constant development of smart grids and deployment of smart meters, the related operation data also significantly increase in terms of volume, velocity, variability and complexity. It leads to a series of challenging problems considering the limited data storage and computing power. To solve such issues, one approach is to apply cloud computing technologies [135]. With the improvement of communication technologies, sophisticated data processing and storage procedures could be realized through cloud services. The cloud-based smart metering model is formed to optimize energy utilization through cloud platforms such as IBM Coremetrics and Google BigQuery [23]. Alternatively, edge computing technologies would also enhance the computing power in a distributed manner [136]. It mitigates the burdens of the centre cloud computing terminals. However, as smart meters often monitor different types of data, it brings about variability and complexity to process them. The smart grid faces the challenge to process mixed data types in different time scales, formats and forms. Particularly for applications related to forecasting and optimization, the smart grid needs to process data from different aspects such as the power networks and climate. Therefore, data standardization and data fusion are required to establish smart grids. In addition, as the operation data are uploaded for cloud computing, it also raises privacy and security issues as the data may be vulnerable to unauthorized usage.

### 4.3. Cooperation with “Smarter” AI

The term AI is represented by intelligent computer programs. With rapid evolution of machine learning technologies and acceleration of hardware improvements, AI-based computer programs can behave more intelligently according to different situations [137]. On the other hand, the interconnected smart meters create the environment of the Internet of Things (IoT) as it facilitates energy interaction among prosumers and significantly increases the capacity of devices number connected to the smart grid [138]. The devices are monitored and managed by the mutually connected smart meters, and they coordinate according to the optimal control signal to realize system-wise corporations. The optimization is provided by the IoT-based smart grids, which is expected to become “smarter” as it opens up possibilities to integrate AI into power systems [139]. The decision making, self-organizing and cognitive functionalities enable the smart grid to obtain comprehensive awareness of system status and act more like a grid operator [140]. Using the measured data from smart meters, the AI algorithms can be trained to be more suitable for handling operations in smart grids. However, there are also challenges considering the limitation of the existing AI algorithms and how humans can better corporate with AI in smart grid operations.

### 4.4. Consumers’ Engagement and Awareness

The engagement of consumers is crucial as they are the end-users of smart meters. Their acceptance of smart meter fundamentally affects the data quality of the smart grid, which further influence its performance. Although smart meters bring about great benefits to the operations of smart grids, consumers may still hesitate to install them due to multiple concerns [141]. Such negative concerns slow down the installation process and hinder the development of smart grids. It is reported that the global implementations of smart meters have been interrupted by obstacles multiple times [142]. To improve the engagement and awareness of consumers, there is a challenge to highlight the importance of smart meters and to make suitable policies to facilitate their implementations. Specifically, the prior question is to understand the user perception of adopting smart meters. Studies find that barriers can be categorized into several factors [143].

1. External and internal influence: The external influence indicates the impact of user’s decisions externally such as policies or other users. In contrast, the internal influence indicates the intrinsic determinant that impacts the user’s decisions, such as awareness and self-motivation. To overcome such barriers, some approaches can be applied, including incentives and publicity.
2. User appeal and ability: User appeal and ability refer to the smart metering technologies that can constantly attract users’ interest and provide suitable control ability for users to manage their power consumption. To overcome such barriers, personalized services can be applied to satisfy individual requirements.
3. Reliability: The reliability of the smart meter is essential to guarantee reliable operations and low maintenance costs. It requires the smart meter to operate under extreme conditions like low temperatures, high voltages and disclosure to electromagnetic waves, while maintaining high performance. High reliability can also improve the customers’ confidence and satisfaction.
4. Ease of use: Ease of use stands for the difficulty of users interacting with smart meters. As most of the smart meters are installed on the consumer side, they are the interface for consumers to interact with smart grids. Therefore, a user-friendly UI of smart meters is important for consumers to better track their energy usage and understand consumption policies to rapidly respond to the variations in smart grids.
5. Privacy: Smart meter data are regarded as users’ privacy, where the level of data security impacts their acceptance of smart meters. Therefore, advanced privacy protection technologies are required.

The factors can be used to guide future energy management system design and policy-making. The proper design would improve the consumers’ knowledge of the energy transition towards renewable energies. It also facilitates the consumers to install suitable peripherals for smart meters to enhance their performance and provide more benefits to consumers. In addition, consumers are less motivated, concerning the expensive initial installation cost of smart meters with limited benefits in return [144]. Improving the performance of operations in smart grids, as well as expanding smart meter implementations with novel applications, would potentially promote their installation benefits. For instance, smart metering technologies can support the fourth industrial revolution, which can be expanded to multiple industry fields [145]. A case study is conducted in Singapore, which investigates the challenges and strategies for adopting smart technologies in the construction industry. The study seeks to enhance industrial performance by improving workflows and work conditions [146].

## 5. Conclusions

In this paper, a comprehensive review of the control and optimization of smart grids using smart meters is presented. Challenges for the energy transition towards renewable energies future are identified in both the smart grid and smart meter perspectives. It is shown that energy providers and consumers in smart grids coordinate with each other through various applications, which are supported by multiple control and optimization technologies. The applications from smart grid and smart meter perspectives are mutually supported and facilitated, which can be summarized in the following ideas.

1. From the smart grid perspective, many applications are developed to facilitate the penetration of renewable energies and to increase the benefit for grid operators and consumers with the support of smart meters. The evolution of communication and computation technologies further pushes the development of the smart grid towards the IoT in the future, where more electric devices are expected to be involved in more intelligent cooperative operations.
2. From the smart meter perspective, the development of smart grids reflects several essential features, which promote the evolution of smart meter applications. The applications are not only dedicated to improving the operation performance, but also enhance the relationship between customers. Customer awareness significantly influences the implementation of smart meters. Requirements such as personalized services and privacy protection stimulate the advancement of smart metering technologies, which are expected to have more interactions with consumers.

## Figures and Tables

**Table 1:** The functionalities of smart grid.

Functionalities	Description
Self-healing capability	The ability of awareness and isolation of operating faults. The capability improves the reliability of the smart grid and provides better service for infrastructure maintenance.
Consumer participation	Consumers can be actively involved in the smart grid to help balance the supply and demand by changing their energy utilization behaviors.
Resilience to attacks	Resilience to cyber and physical attacks and natural catastrophes; quick recovery from a disturbance.
Power quality	Power quality is appropriate for modern society. It includes real-time monitoring and control techniques for diagnosing and solving issues that impact power quality.
Integration of multiple generation	Integration of multiple distributed renewable energy resources.
Interactive with the market	The bidirectional communication at various sections of the grid enables a better atmosphere for the power trading market. The prosumers could alter their energy consumption and production by choosing competing services.
Asset maintenance	Condition-based asset maintenance aiming at minimizing the influence on consumers.

## Data Availability

Not applicable.
